# Supplementary material for: GRIDSS2: comprehensive characterisation of somatic structural variation using single breakend variants and structural variant phasing
Source: Genome Biol. 2021 Jul 12;22:202. doi: 10.1186/s13059-021-02423-x (PMC8274009; doi:10.1186/s13059-021-02423-x)
Supplement: Supplementary file 1 — Additional file 1. Fig.S1-S4. Fig. S1 COLO829 results with/without PON filter. Fig. S2 PCAWG/GRIDSS, and PURPLE/PCAWG SV/CNV consistency. Fig. S3 PCAWG single breakend annotations. Fig. S4 Phasability and LINX classification for Hartwig and PCAWG. [file 13059_2021_2423_MOESM1_ESM.pdf]

GRIDSS2 Supplementary Figures

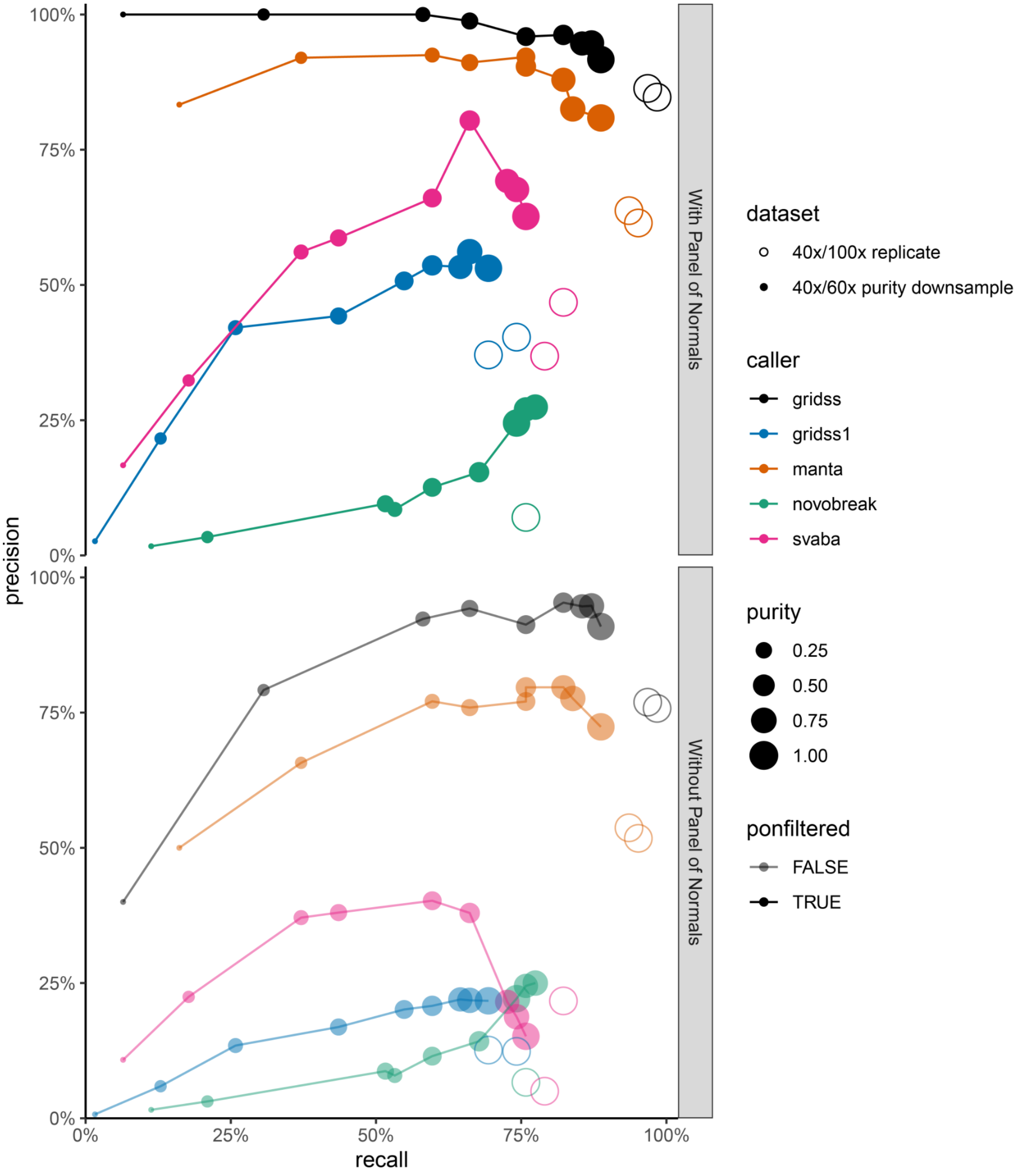

Figure S1: COLO829 results with and without filtering with the GRIDSS2 panel of normals.

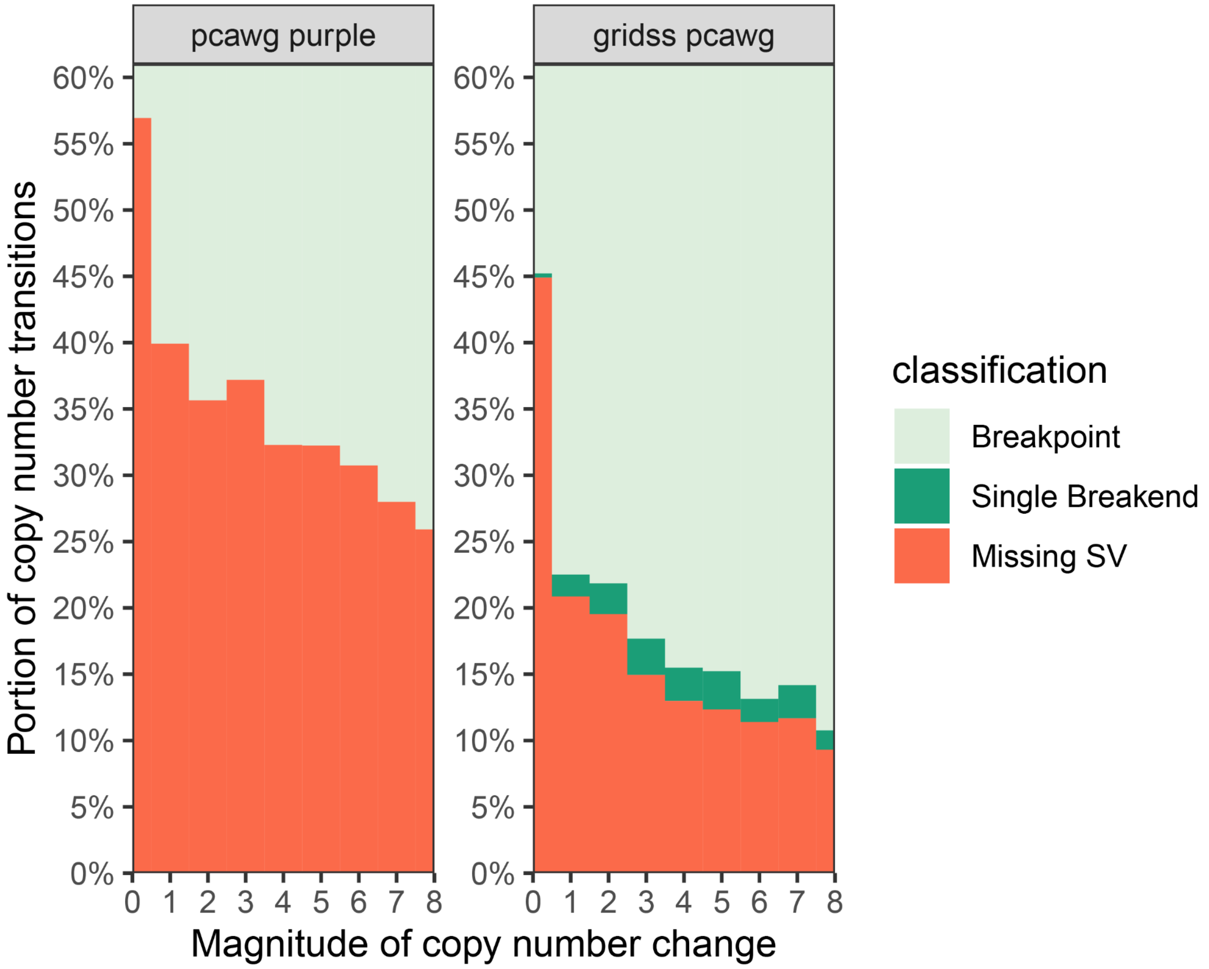

Figure S2: Structural variant consistency on the PCAWG cohort when pairing the PCAWG consensus SV calls with PURPLE CNVs, and GRIDSS2 SV calls with the PCAWG consensus CNVs.

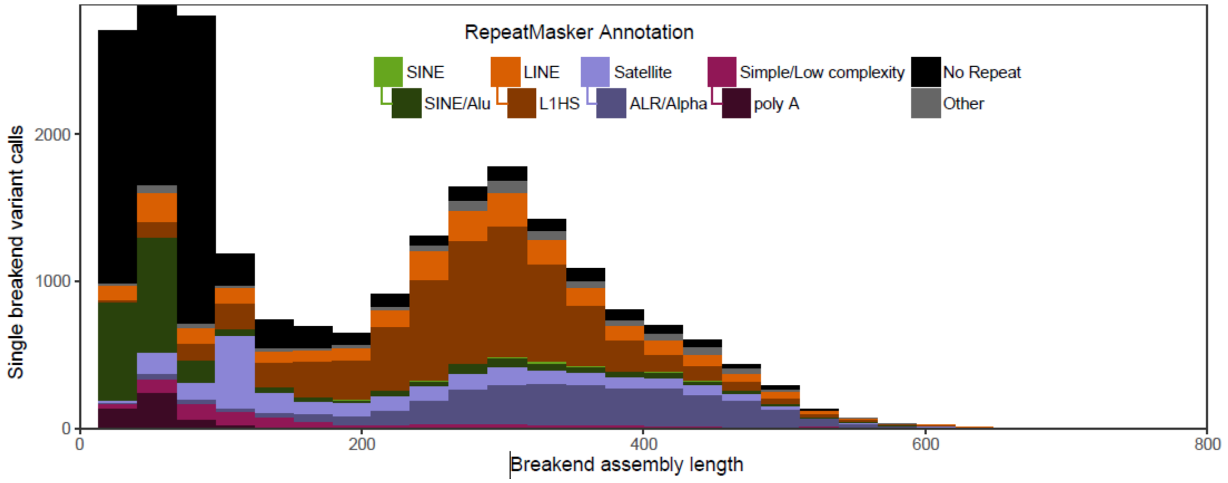

Figure S3: Single breakend RepeatMasker annotation for 1,476 PCAWG samples.

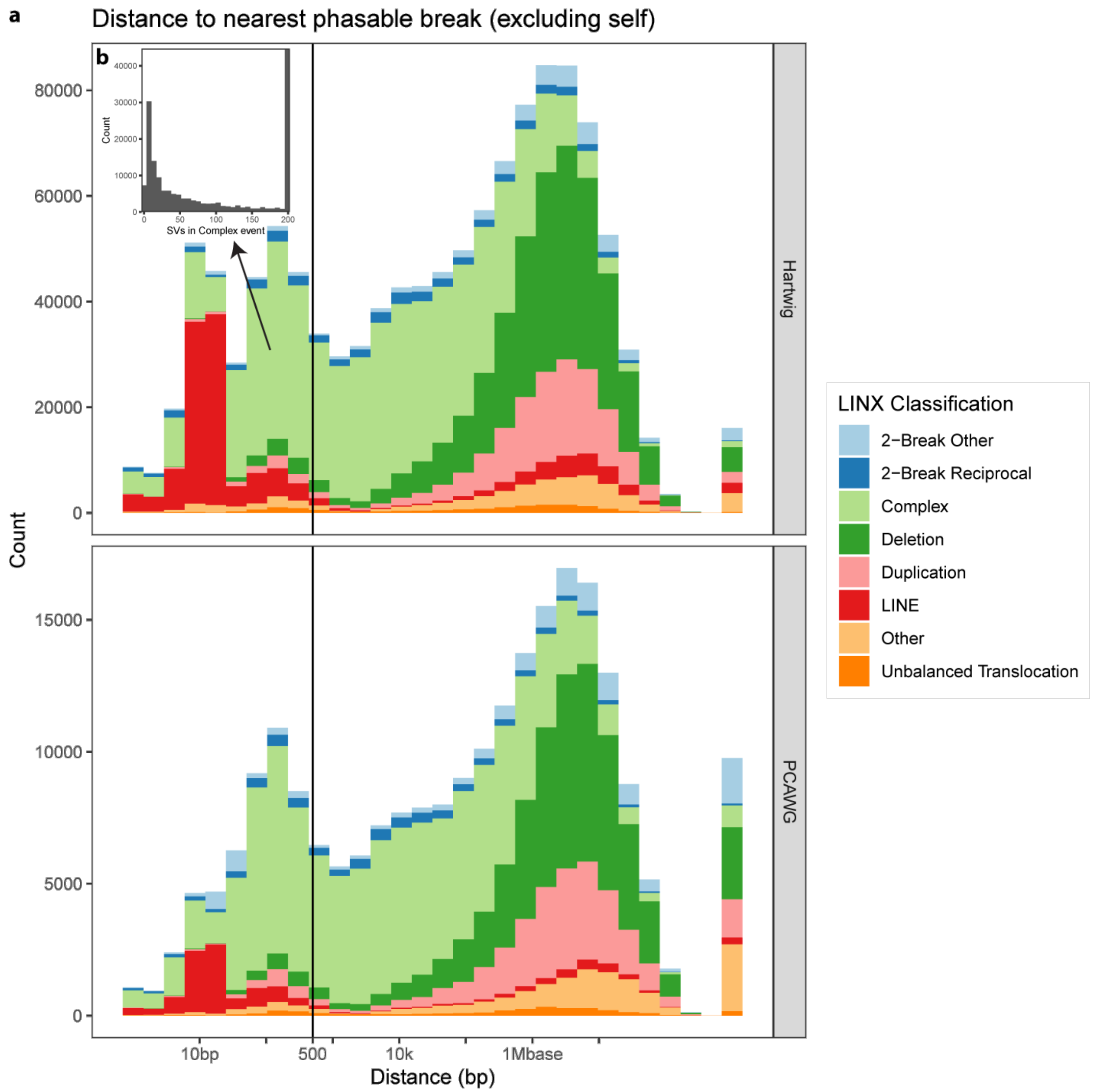

Figure S4: a) Phasability and LINX classification of structural variants in the Hartwig and PCAWG cohorts. Phasable variants are predominantly LINE translocation or form part of complex events. b) Number of SVs in complex event clusters containing phasable SVs. Most phasable SVs occur in highly complex events.
