# Supplementary material for: GRIDSS2: comprehensive characterisation of somatic structural variation using single breakend variants and structural variant phasing
Source: Genome Biol. 2021 Jul 12;22:202. doi: 10.1186/s13059-021-02423-x (PMC8274009; doi:10.1186/s13059-021-02423-x)
Supplement: Supplementary file 3 — Additional file 3. Review history. [file 13059_2021_2423_MOESM3_ESM.docx]

Review History

**First round of review**

**Reviewer 1**

**Are you able to assess all statistics in the manuscript, including the appropriateness of statistical tests used?**

**Yes: NA**

**Comments to author:**

The authors described the algorithm of GRIDSS2 with 2 key steps: assembly and phasing. They showed the better performance of GRIDSS2 using benchmark "gold standard" somatic SV call set for the COLO829 melanoma cell line and the COLO829BL cell line with 3 technical replicates of real data, as well as the simulated lower sequencing depth data compared to other callers. In addition, they also performed experimental validation using the patient samples from the Hartwig cohort with a higher validation rate compared to Manta and Strelka (smaller indels). The feature of GRIDSS2 to identify somatic SVs by single breakend into repeats and centromere region could resolve the somatic SVs which were missed by previous methods. In the end, they also showed that by phasing the SVs identified, GRIDSS2 is able to resolve some complex SV events.

Overall, the manuscript is well organized and has shown how GRIDSS2 is different from previous algorithms.

Here are some minor points about the figures:

1. In Figure 1b, it would better to state that different grey colors represent different haplotypes phased based on junction sequences in the figure caption.

2. In Figure 2d, it seems not clear where the PURPLE result is. It seems like the left panel should be the result of PURPLE, but it is mis-labeled.

3. In Figure 3a, the x-axis legend ("Breakend assembly length") is too far away from the figure but closer to 3c, which could be confusing.

4. In Figure 3d, it seems like chromosome 8 also have high level of both intra and inter chromosomal events, but it is not discussed or mentioned.

I also have some additional questions about the result:

1. What is the size distribution of the CNVs the authors performed validation on with the Hartwig cohort (Figure 2b)? Is there any bias towards smaller or larger event which were validated? Are there any single end breakpoints to centromere/repeats that are validated here. (These information may be included in Supplementary table 1, but I did not find the file.)

2. Can GRIDSS2 also find events with single breakends which have the other end map to telomere?

**Reviewer 2**

**Are you able to assess all statistics in the manuscript, including the appropriateness of statistical tests used?**

**Yes: I was able to assess all statistics in the manuscript and the tests used were appropriate.**

**Comments to author:**

The authors present GRIDSS2 which is a second version of a structural variation (SV) caller that now accounts for single breakend variants. Existing SV callers do not predict single breakend events due to breakpoints being ambiguously located in regions, often repetitive, that are difficult to map using short reads. The authors demonstrate the utility of GRIDSS2 and its single breakend calling on various WGS datasets including deeply characterized, gold-standard melanoma cell lines, PCAWG/ICGC pan-cancer cohorts, and the Hartwig Medical Foundation pan-cancer data from metastatic solid tumors. They show that GRIDSS2 out-performs existing tools when calling short (32-50bp) and, overall, results in a 3.1% rate of unexplained copy number (CN) transitions. The authors demonstrate that 7% of CN transitions can only be explained by single breakend variants. Further, they show that single breakends are enriched in centrmeric sequences, with 47% coincident with CN transitions, and can be mapped to centromeres on multiple chromosomes. Finally, SV phasing by GRIDSS2 highlights LINE translocations and can be used for complex rearrangement analysis with low false negative rates.

This study presents an interesting and bold perspective on an SV type that has been largely omitted by existing SV callers due to the challenge to confidently resolve unambiguously. If the prediction of single breakends are real and the claims are justified, then GRIDSS2 will be useful for cancer researchers. However, there are several concerns that may potentially dampen the enthusiasm or limit the novelty of this study. In general, my concerns are focused on the lack of strong evidence or validation that single breakends are not artifacts.

Major Comments:

1. Benchmarking performance section

a. A panel of normal (PON) is described for GRIDSS2 and I assume was used for performance evaluation against the benchmark (COLO829). The use of a PON can be quite useful both for biological, technical, and algorithmic artifacts. The other tools in the comparison may not be using a PON but it is trivial/routine to include as a final filter generated from the same tool in the evaluation and would provide a much more reasonable comparison. This may address the high false positives (low precision) of the other tools.

b. It would be informative to compare GRIDSS vs GRIDSS2 to see how the newer version improves on performance.

2. Validation on patient samples

a. Single breakend variant probes appear to be included in the capture panel (according to the methods). What is the performance of GRIDSS2 for predicting single breakends?

b. What was the validation performance for short duplications (32-100bp)? The claim is that this is a novel somatic signature and should be validated, especially if the capture panel includes this data.

3. FNR using copy number consistency

a. How were the PCAWG samples chosen? Only 1528 samples are included out of the possible 2500+ samples - what is the justification?

b. How was the PCAWG consensus call set used? The SVs in this set contains information on which tool supported the prediction and sometimes multiple tools are noted, which is how the consensus was determined. When >1 supporting tools criteria was used, it promoted higher specificity but may explain the lower sensitivity (and higher FNR) in your comparison. The same question applies to the PCAWG CNV consensus calls.

4. A major concern is the use of CN transitions as the benchmark for determining FNR. CN transitions are themselves predicted by PURPLE and compiled by PCAWG consensus CNVs. What is the performance (FNR) of GRIDSS2 when comparing to PCAWG CN transitions? How does PCAWG SV calls compare to PURPLE CN transitions?

5. Is it at all possible that other tools can still make predictions involving breakpoints at locations that are considered single-breakend calls by GRIDSS2? How do the GRIDSS2 single breakend event sites compare with predictions from other tools?

6. Novel centromeric break signature. The claim that this is a novel signature appears to be not well-supported. The centromeres at chrs 1,5,19 have high sequence similarity. The justification with CN transitions in centromeres is also problematic because CN events at these regions also potentially suffer from issues of mappability and repetitive sequences that may affect coverage.

Minor Comments:

1. Figure 2d: Is "60" PCAWG samples correct? The figure lists 1528 samples.

2. Line 159: Figure 2f should be 2g?

3. Line 163: Figure 2g should be 2f?

4. Line 164: "Across the Harwig cohort, 23 samples …" - should have a reference to a table or figure. Also, why are these drivers? What are the genes?

5. Line 350: "b)" should be "c)"

**Authors Response**

**Point-by-point responses to the reviewers’ comments:**

**Reviewer #1:**

The authors described the algorithm of GRIDSS2 with 2 key steps: assembly and phasing. They showed the better performance of GRIDSS2 using benchmark "gold standard" somatic SV call set for the COLO829 melanoma cell line and the COLO829BL cell line with 3 technical replicates of real data, as well as the simulated lower sequencing depth data compared to other callers. In addition, they also performed experimental validation using the patient samples from the Hartwig cohort with a higher validation rate compared to Manta and Strelka (smaller indels). The feature of GRIDSS2 to identify somatic SVs by single breakend into repeats and centromere region could resolve the somatic SVs which were missed by previous methods. In the end, they also showed that by phasing the SVs identified, GRIDSS2 is able to resolve some complex SV events.

Overall, the manuscript is well organized and has shown how GRIDSS2 is different from previous algorithms.

Here are some minor points about the figures:

1. In Figure 1b, it would better to state that different grey colors represent different haplotypes phased based on junction sequences in the figure caption.

*The transitive links at the bottom of figure 1b are not haplotypes, but false positive transitives breakpoints calls. These transitive false positives are present in short read data whenever two breakpoints are closer together than the library fragment size distribution. That is, when the genome contains a rearrangement resulting in DNA segments A-B-C (thus having cis-phased breakpoints A-B, and B-C), a false positive A-C breakpoint call will be made when B is small. When B is small, a short read caller will report precise A-B, and B-C breakpoints, but will also report an imprecise A-C breakpoint because there will be some fragments that completely span B and have a read aligning to A and a read aligning to C. The A-C call is imprecise since it’s supported only by discordant read pairs. (See red read pairs in the updated Fig1b).*

*This results in a short read caller reporting A-B, B-C, and A-C breakpoint calls. When there are two small fragments (as in the fig1 example), an A-B-C-D rearrangement results in precise A-B, B-C, C-D calls, but also imprecise A-B, A-D, B-D calls.*

*We found that the presence of these imprecise transitive calls is extremely problematic when performing complex event reconstruction. Adding a transitive call reduction step to GRIDSS2 significantly improves the complex event reconstruction capabilities of our downstream tool (https://www.biorxiv.org/content/10.1101/2020.12.03.410860v1) as not only does it remove these false positive call which are inconsistent with the correct interpretation of the actual rearrangement, but it provides an additional phasing signal.*

*This approach is similar to our earlier work where we showed that optical mapping gaps can be explained by traversing multiple GRIDSS breakpoint calls (PMID 29618486).*

*A similar issue occurs with long read callers. When B is shorter than the alignable length, then the long read caller will report an A-C breakpoint with the inserted sequence of B. As we mention in our discussion, this is a real issue when performing somatic joint short+long read calling since the short read and long read callers will both detect the same rearrangement, but they will report it differently and this must be taken into account. The highly clustered nature of somatic SVs means that these notational differences are actually very common.*

*● Fig 1b text has been expanded to “Read pairs spanning multiple breakpoints causes false positive imprecise transitive calls. These are filtered and replaced by cis phasing of precise calls”*

*● Fig1b: Transitive calls and their corresponding read pairs are now coloured red to highlight that they are problematic.*

*● We have added the following to the discussion:*

*“GRIDSS2’s ability to collapse imprecise transitive calls into their corresponding precise breakpoints is similarly essential to complex event reconstruction as these transitive calls result in spurious false positives that are inconsistent with the actual rearrangement structure.”*

2. In Figure 2d, it seems not clear where the PURPLE result is. It seems like the left panel should be the result of PURPLE, but it is mis-labeled.

*Fig 2de are SV/CN consistency results thus the plots are the combination of both an SV caller, and a CN caller. Fig2d left is PCAWG consensus SV + PCAWG consensus CNVs and Fig2e is GRIDSS2 SVs + PURPLE CNVs.*

*● We have relabeled the Fig2de labels as GRIDSS2/PURPLE.*

3. In Figure 3a, the x-axis legend ("Breakend assembly length") is too far away from the figure but closer to 3c, which could be confusing.

*Agreed - that was definitely put in the wrong place.*

*● We have moved the label closer to the figure.*

4. In Figure 3d, it seems like chromosome 8 also have high level of both intra and inter chromosomal events, but it is not discussed or mentioned.

*We have dropped our centromeric rearrangement signature result as we do not have sufficient orthogonal SKY FISH or optical mapping validation data to definitively demonstrate the validity of our matching centromere predictions.*

I also have some additional questions about the result:

1. What is the size distribution of the CNVs the authors performed validation on with the Hartwig cohort (Figure 2b)?

*Figure 2b is a comparison of all breakpoints (or single breakends) in which the two constituent breakpoints are at least 50bp apart. This include inter-chromsomal breakpoints and there is not necessarily any copy number change associated with the breakpoints (e.g. a true inversion will have two breakpoints but no associated CNV).*

Is there any bias towards smaller or larger event which were validated?

*There is no bias within the samples as to which SVs were chosen for validation as we generated gaptures probes were generated for all SVs >=32bp in the 13 samples. The samples chosen were samples that 1) had a lot of SVs, and 2) appeared to have an overabundance of small duplications. This enables us to not only do our gridss/manta/strelka comparison, but also to validate whether the novel small duplication signature we found was actually real. Since the choice of samples was biased towards those with more SVs (so we had more variants to do the validation on), then there will be some sample selection bias towards rearrangement events/signatures that result in many SV calls (e.g. chromothripsis, LINE reactivation).*

*Although there are some specific features of interest (e.g. interchromosomal and single breakend GRIDSS calls have lower validation rates), the validate rate comparison broadly holds across the spectrum of SVs, and the spectrum has a similar overall shape and distribution as the Hartwig cohort overall. For these reasons, we believe that the validation results are broadly representative of the Hartwig cohort overall.*

Are there any single end breakpoints to centromere/repeats that are validated here?

*● We have added the following result to the “Validation on patient samples” section:*

*○ “89% (170 of 192) GRIDSS single breakend calls were validated, with 17 of the 22 failing validation occurring in simple repeats. 98% (53 of 54) of centromeric and 88% (61 of 69) of LINE/L1 single breakends passed validation indicating robust single breakend performance outside of simple repeats.”*

(These information may be included in Supplementary table 1, but I did not find the file.)

*Apologies for this oversight.*

*● We have now included Supplementary Table 1 in our submission*

2. Can GRIDSS2 also find events with single breakends which have the other end map to telomere?

*Yes. We have identified a telomeric rearrangement signature and associated driver gene but will be publishing this result elsewhere.*

Reviewer #2:

The authors present GRIDSS2 which is a second version of a structural variation (SV) caller that now accounts for single breakend variants. Existing SV callers do not predict single breakend events due to breakpoints being ambiguously located in regions, often repetitive, that are difficult to map using short reads. The authors demonstrate the utility of GRIDSS2 and its single breakend calling on various WGS datasets including deeply characterized, gold-standard melanoma cell lines, PCAWG/ICGC pan-cancer cohorts, and the Hartwig Medical Foundation pan-cancer data from metastatic solid tumors. They show that GRIDSS2 out-performs existing tools when calling short (32-50bp) and, overall, results in a 3.1% rate of unexplained copy number (CN) transitions. The authors demonstrate that 7% of CN transitions can only be explained by single breakend variants. Further, they show that single breakends are enriched in centrmeric sequences, with 47% coincident with CN transitions, and can be mapped to centromeres on multiple chromosomes. Finally, SV phasing by GRIDSS2 highlights LINE translocations and can be used for complex rearrangement analysis with low false negative rates.

This study presents an interesting and bold perspective on an SV type that has been largely omitted by existing SV callers due to the challenge to confidently resolve unambiguously. If the prediction of single breakends are real and the claims are justified, then GRIDSS2 will be useful for cancer researchers. However, there are several concerns that may potentially dampen the enthusiasm or limit the novelty of this study. In general, my concerns are focused on the lack of strong evidence or validation that single breakends are not artifacts.

Major Comments:

1. Benchmarking performance section

a. A panel of normal (PON) is described for GRIDSS2 and I assume was used for performance evaluation against the benchmark (COLO829). The use of a PON can be quite useful both for biological, technical, and algorithmic artifacts. The other tools in the comparison may not be using a PON but it is trivial/routine to include as a final filter generated from the same tool in the evaluation and would provide a much more reasonable comparison. This may address the high false positives (low precision) of the other tools.

*We have now split out the GRIDSS PON filter and address it explicitly:*

*● Figure 2 now applies the GRIDSS PON to all callers*

*● We have added a supplementary figure showing performance of all callers without a PON applied*

*● Our simulation results now reads:*

*○ “With the GRIDSS panel of normals (PON) filter applied, GRIDSS2 achieved an average sensitivity/precision of 98%/86% compared to 94%/63% for Manta, 72%/39% for GRIDSS1, 81%/42% for svaba and 76%/7% for novobreak (Figure 2a). Without the PON, precision was reduced to 76%, 53%, 13%, 13%, 7% for GRIDSS2, Manta, GRIDSS1, svaba, and novobreak respectively”.*

b. It would be informative to compare GRIDSS vs GRIDSS2 to see how the newer version improves on performance.

*● We have added GRIDSS1 to the COLO829 comparison.*

2. Validation on patient samples

a. Single breakend variant probes appear to be included in the capture panel (according to the methods). What is the performance of GRIDSS2 for predicting single breakends?

*● We have added the following result to the “Validation on patient samples” section:*

*○ “89% (170 of 192) GRIDSS single breakend calls were validated, with 17 of the 22 failing validation occurring in simple repeats. 98% (53 of 54) of centromeric and 88% (61 of 69) of LINE/L1 single breakends passed validation indicating robust single breakend performance outside of simple repeats.”*

b. What was the validation performance for short duplications (32-100bp)? The claim is that this is a novel somatic signature and should be validated, especially if the capture panel includes this data.

*384 of 386 GRIDSS 32-100bp DUP calls validated. There were 2 small duplications called by manta that validated but were missed by GRIDSS2.*

*● We have added a Figure 2e: manta/gridss/strelka small duplication validation results.*

3. FNR using copy number consistency

a. How were the PCAWG samples chosen? Only 1528 samples are included out of the possible 2500+ samples - what is the justification?

*Our choice of samples is based on the following criteria:*

*- ICGC sample (we did not have TCGA data access)*

*- Passing PURPLE quality control checks (samples with known copy number issues are poor candidates for using CN as an orthogonal validation of breakpoints).*

*- Only 1 sample for that donor.*

*- Unfortunately, our analysis pipeline saved results using donor name not sample GUID. This means we can’t disambiguate which sample the result came from so we have to drop all samples for those donors.*

*Since initial submission, we have rerun on the latest version of GRIDSS2/PURPLE which has additional QC checks. We now have the following sample filtering breakdown*

*- 1839 ICGC samples*

*- 1719 after removing donors with multiple specimens sequenced*

*- 1698 after removing missing PCAWG SV consensus calls*

- 1476 after removing PURPLE QC not PASS

*● We have updated the results to reflect the results from the 1476 sample subset*

*● We have updated the methods to include the sample filtering criteria*

b. How was the PCAWG consensus call set used? The SVs in this set contains information on which tool supported the prediction and sometimes multiple tools are noted, which is how the consensus was determined. When >1 supporting tools criteria was used, it promoted higher specificity but may explain the lower sensitivity (and higher FNR) in your comparison. The same question applies to the PCAWG CNV consensus calls.

*The SV and CNV calls were directly taken from the publicly available PCAWG consensus call sets with no post-processing performed. The SV and CNV consensus calls were downloaded from:*

*https://dcc.icgc.org/releases/PCAWG/consensus_sv/final_consensus_sv_bedpe_passonly.icgc.public.tgz*

*https://dcc.icgc.org/releases/PCAWG/consensus_cnv/consensus.20170119.somatic.cna.icgc.public.tar.gz*

*The *consensus.*.somatic.cna.txt CNV files downloaded from above did not include any information about the callers used to generate the consensus CNV calls and were taken as is.*

*The *.pcawg_consensus_*.somatic.sv.bedpe did include a “svmethod” column but this column was ignored in analysis. Our analysis was based entirely on the publicly available BEDPE file. If a call was included in the final_consensus_sv_bedpe_passonly.*public.tgz official final consensus SV call set, we included it in our analysis. As we did not perform any SV call filtering, the higher FNR of the PCAWG consensus SV call set must be due to a combination of the filtering criteria the PCAWG consortium used to generate their consensus call set, and the comprehensiveness of the callers used in their ensemble calling. That GRIDSS2 is even comparable to the performance of the PCAWG consensus call set is a clear demonstration of the excellent somatic SV calling performance of GRIDSS2.*

*One issue we encountered is that the PCAWG CN consensus reports integer CNs. Since we were unsure of whether the final SV consensus included subclonal SVs, we have provided a breakdown by CN transition magnitude in our plots, and performed the FNR analysis both with and without restricting to clonal events (ΔCN>=1).*

4. A major concern is the use of CN transitions as the benchmark for determining FNR. CN transitions are themselves predicted by PURPLE and compiled by PCAWG consensus CNVs. What is the performance (FNR) of GRIDSS2 when comparing to PCAWG CN transitions? How does PCAWG SV calls compare to PURPLE CN transitions?

*We have expanded our PCAWG comparison to include these results.*

*● “As both the PCAWG consensus CNV and PURPLE copy number segmentation are, in part, driven by the PCAWG consensus SV and GRIDSS2 calls, there is the potential for the copy number segmentation to be over-optimised for the corresponding call set. To evaluate the extent of this, we compared the GRIDSS2 SV call sets to the PCAWG consensus CNV, and the PCAWG consensus SV to the PURPLE CNV calls. In both cases, FNR increased with PCAWG/PURPLE at 40.7% and GRIDSS2/PCAWG at 33.8%. Restricting to clonal copy number transitions GRIDSS2/PCAWG FNR reduced to 19.4% whereas PCAWG/PURPLE remained high at 38.3% (Supplementary Figure 2). Examining the copy number transitions not explained by their matching SV caller, we found the PCAWG consensus SV call set includes explanatory SVs at 5,928 PURPLE copy number transitions that were missed by GRIDSS2. This is less than half of the 9,433 PCAWG copy number transitions explained by GRIDSS2 (2,415 by single breakends) but not the PCAWG consensus SVs - an indication that the GRIDSS2 call set is likely to be more comprehensive than the PCAWG consensus SV calls.”*

5. Is it at all possible that other tools can still make predictions involving breakpoints at locations that are considered single-breakend calls by GRIDSS2? How do the GRIDSS2 single breakend event sites compare with predictions from other tools?

*We have added a new “Single breakend performance” results section in which we simulate breakpoints (half to centromeric sequence) from the complete telomere-2-telomere CHM13 chr8 reference. To reduce the computational cost of performing ~3000 simulations, we used only chr8 as a reference, and only simulated the 10kbp around breakpoint. For GRIDSS2/manta/svaba we also included in the BAM an additional 2,000,000 reads to ensure their library fragment size distribution calculation was correct (svaba requires 1M read pairs)^.*

*This represents an ‘easy’ yet relatively realistic simulation of centromeric breakpoints. It is a useful best-case comparison of the tools’ detection capabilities.*

*We found that the manta and svaba were blind to centromeric integration due to their reliance of read mapping; that novobreak’s approach of direct kmer matching enable it to detect most centromeric integrations but would report multiple integrations 32% of the time; and that GRIDSS2’s single breakend variant calling approach could reliably detect of somatic centromeric integrations without overcalling.*

*These results are consistent with the benchmarking results from VIRUSBreakend (https://doi.org/10.1093/bioinformatics/btab343), our viral integration detection tool that uses GRIDSS2 single breakends against a viral genome to detect integrations. VIRUSBreakend’s usage of GRIDSS2 under to hood allows it to reliably detect viral integration in repetitive regions (such as centromeres) whereas the other viral integration detection tools would over or under count viral integrations in such regions.*

*^ Since these reads were added to both the tumour and normal BAMs, their inclusion does not impact the somatic calling results. Every one of these in the tumour has an exactly matching read in the normal (since it’s the same BAM file being merged into both the tumour and the normal).*

6. Novel centromeric break signature. The centromeres at chrs 1,5,19 have high sequence similarity. The justification with CN transitions in centromeres is also problematic because CN events at these regions also potentially suffer from issues of mappability and repetitive sequences that may affect coverage. The claim that this is a novel signature appears to be not well-supported.

*The 1,5,19 centromeres do indeed have high sequence similarity. For this reason Figure 3c was restricted to only the subset of centromeres with both a copy number change, and a single explanatory single breakend call. We agree that mappability and copy number instability is an issue in the pericentromeric regions. This is mitigated by performing pair tumour/normal CN calling, and by the centromere-specific smoothing logic included in PURPLE specifically to address this issue. That said, it is true that we have not presented truely orthogonal validation data for our centromeric predictions.*

*Unfortunately, centromeric SVs are particularly difficult to obtain orthogonal validation data for as even long read sequencing is unable to reliably fully span centromeres. SKY FISH (spectral karyotyping) or optical mapping data would be suitable but unfortunately we do not have such data available for our cohort. We have removed our claim of finding a novel centromeric break signature from this manuscript.*

*● Our novel centromeric break signature results have been removed*

Minor Comments:

1. Figure 2d: Is "60" PCAWG samples correct? The figure lists 1528 samples.

*60 samples was correct for an earlier revision of the manuscript, but not the submitted manuscript. The figure legend was not updated when the results were expanded to 1,528 samples.*

*● Fig2d legend text has been fixed.*

2. Line 159: Figure 2f should be 2g?

*It should.*

*● Figure references have been updated.*

3. Line 163: Figure 2g should be 2f?

*It should.*

*● Figure references have been updated.*

4. Line 164: "Across the Harwig cohort, 23 samples …" - should have a reference to a table or figure. Also, why are these drivers? What are the genes?

*Driver status was determined as outlined in Priestley et al 2019.*

*● We have added a citation to the driver status determination logic.*

*● We have rephrased “23 samples” to “21 patients” as two of the patients with small dup drivers were sequenced a second time due to relapse.*

*● We have added supplementary table 2 which contains the small duplications predicted to be drivers (including the relevant driver gene).*

5. Line 350: "b)" should be "c)".

*It should.*

*● Figure references have been updated.*

**Second round of review**

**Reviewer 1**

**The authors have addressed all my questions and concerns. And I am looking forward to the telomeric rearrangement in their future publication.**

**Reviewer 2**

**The authors have addressed all of my concerns.**
